# Modified hybrid cementing technique reduces stem tip pain and improves patient’s satisfaction after revision total knee arthroplasty

**DOI:** 10.1186/s13018-020-01921-1

**Published:** 2020-09-09

**Authors:** Man Soo Kim, In Jun Koh, Sueen Sohn, Hyung Chul Park, Yong In

**Affiliations:** 1 Department of Orthopaedic Surgery, Seoul St. Mary’s Hospital, College of Medicine, The Catholic University of Korea, 222, Banpo-daero, Seocho-gu, Seoul, 06591 Republic of Korea; 2 Department of Orthopaedic Surgery, The Catholic University of Korea Graduate School, 222, Banpo-daero, Seocho-gu, Seoul, 06591 Republic of Korea; 3 Department of Orthopaedic Surgery, Eunpyeong St. Mary’s Hospital, College of Medicine, The Catholic University of Korea, 1021, Tongil Ro, Eunpyeong-gu, Seoul, 03312 Republic of Korea

**Keywords:** Revision total knee arthroplasty, Satisfaction, Stem tip pain, Cementing technique

## Abstract

**Background:**

There have been no studies comparing patient-reported outcome measures including end-of-stem tip pain and patient satisfaction based on the use of cementing techniques in revision total knee arthroplasty (TKA). The purpose of this study was to compare end-of-stem tip pain and PROMs with hybrid and modified hybrid cementing techniques in revision TKAs.

**Method:**

Sixty-two cases of revision TKA performed by a single surgeon were divided into two groups based on the cementing technique with a minimum follow-up of 2 years. Two types of cementing technique for femoral and tibial stems were used as follows: (1) a hybrid cementing technique (33 cases), in which cement was applied immediately distal to the modular junction of the stem and the component while the distal stem was press-fitted into the diaphysis without using cement; and (2) a modified hybrid cementing technique (29 cases), in which cement was applied to the tip of femoral and tibial stems. The thigh and shin were assessed for the end-of-stem tip pain. Patient satisfaction was evaluated based on the satisfaction items of New Knee Society Score.

**Results:**

Modified hybrid cementing significantly lowered the percentage of patients manifesting shin pain (3.4% vs. 24.2%, *p* = 0.029). Patients treated with the modified hybrid cementing technique showed a higher satisfaction rate (*p* = 0.003). Multivariate logistic regression analysis showed an increase in the odds of satisfaction 32.686-fold (*p* = 0.004) in patients without pain at the end-of-stem tip in the shin and 9.261-fold (*p* = 0.027) in patients treated with the modified hybrid cementing technique.

**Conclusion:**

The modified hybrid cementing technique for fixation of long-stem in revision TKAs reduced the end-of-stem tip pain in the shin, leading to significantly higher satisfaction compared with the hybrid cementing technique after revision TKA.

**Level of evidence:**

Level III

## Background

Revision total knee arthroplasty (TKA) is more difficult than primary TKA due to severe bone defects and insufficient soft tissue [1, 2]. Stem use is necessary in most revision TKAs to facilitate load transmission from articular and metaphyseal bone to the tibial cortex and distribute the increased joint stress [3]. Multiple options are available for stem length and cementation techniques [3, 4]. Metaphyseal fixation utilizes a short stem and diaphyseal fixation uses a long stem [5]. Diaphyseal fixation with a long stem is preferred in most cases because of improved joint stability following increase in stem length and width [3]. The traditional cementing techniques for stem fixation include total and hybrid cementing strategies [4, 6, 7]. In the hybrid cementing technique, cement is applied to the interface between the metaphyseal component and the stem. In the total cement technique, cement is applied to the whole stem in addition to the component [4, 6, 7]. Both methods are associated with their own advantages and disadvantages [4, 6, 7]. Since the total cementing technique is difficult to remove, the hybrid cementing technique is generally preferred for revision TKAs except for a few cases that require total cementation [4]. However, diaphyseal fixation with a long stem is associated with pain at the end-of-stem tip, which might be related to patient dissatisfaction after revision TKA [8, 9]. We have encountered end-of-stem tip pain when using the hybrid cementing technique with long stem for diaphyseal fixation. To address this limitation, we designed a modified hybrid cementing technique for additional cementation at the stem tip.

To the best of our knowledge, there have been no studies comparing PROMs (patient-reported outcome measures) including end-of-stem tip pain and patient satisfaction based on the use of cementing techniques in revision TKAs. The purpose of this study was to compare end-of-stem tip pain and PROMs with hybrid and modified hybrid cementing techniques in revision TKAs. Our hypothesis was that patients treated with the modified hybrid cementing exhibited a lower incidence of end-of-stem tip pain and higher satisfaction after revision TKA compared with patients treated with the hybrid cementing technique.

## Patients and methods

**Fig. 1:**
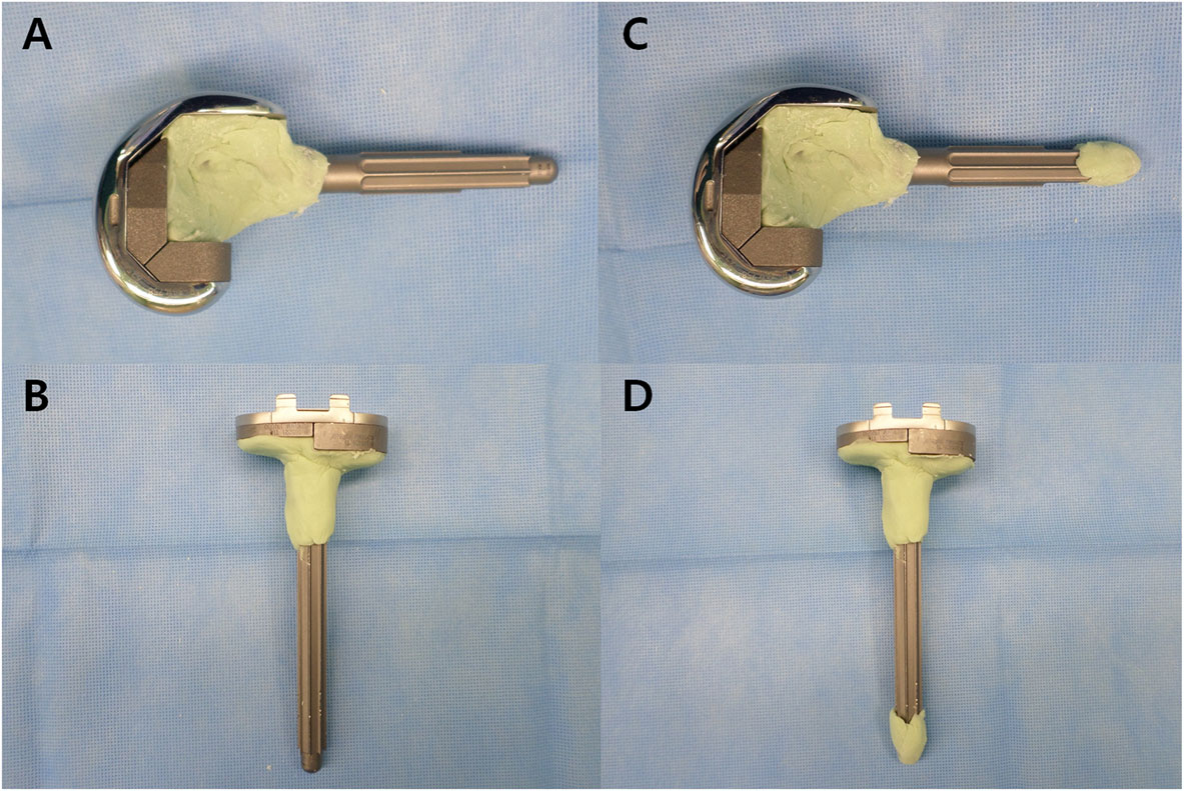
Two types of cementing technique for femoral and tibial stem fixation. With the hybrid technique, the cement was applied around the implant immediately distal to the modular junction of the stem and the component while the distal stem was press-fitted into the diaphysis without cement (**a**, **b**). With the modified hybrid cementing technique, in addition to the hybrid technique, the cement was also used at the tip of femoral and tibial stems (**c**, **d**)

Clinical and radiological data including patients’ charts and radiographs obtained between March 2012 and December 2016 were retrospectively reviewed. All patients who underwent revision TKA were included in this review. A total of 81 consecutive revision TKAs using the same Vanguard 360 revision knee system (Zimmer Biomet, Warsaw, IN, USA) with diaphyseal stem fixation were performed by the senior surgeon at a single institution. We used two types of cementing techniques for femoral and tibial stems. Between March 2012 and March 2015, 41 revision TKAs were performed using the hybrid cementing technique, in which cement was applied around the implant immediately distal to the modular junction of the stem and the component while the distal stem was pressed to fit into the diaphysis without cement [6] (Fig. 1). Starting in April 2015, 40 revision TKAs were performed using a modified hybrid cementing approach, in which the cement was not only applied to the component and modular junction, but also to the tip of the femoral and tibial stem (Fig. 1). The intramedullary canal was prepared by sequential reaming to the appropriate length and diameter to accommodate the press-fit stem. Finally, a stem with a diameter of 1 mm thinner than the press-fit stem size was chosen for stem tip cementing. A finger technique was used for cementation, including the stem tip. Cement was also packed along the prepared bone surfaces. Patients requiring revision TKA only in the femur or tibia and those who were not followed-up 2 years after operation were excluded. In addition, patients who underwent septic revision TKAs, known to be less satisfactory than aseptic revision TKAs [10], were excluded to avoid confounding factors. Two cases undergoing hybrid cementing and one case exposed to modified hybrid cementing were lost to follow-up. The revision of femoral component alone involved a single case in the hybrid cementing group and 2 cases in the modified hybrid group. Thirteen cases (5 in the hybrid group and 8 in the modified hybrid group) underwent two-stage revisions with antibiotic cement spacer for deep periprosthetic joint infection. Therefore, 62 knees were included in the final analysis, including 33 using the hybrid cementing technique and 29 exposed to the modified hybrid cementing technique. This study was approved by the Institutional Review Board (IRB) of our hospital.

The following valuables were compared between the two groups: age, gender, body mass index (BMI), the American Society of Anesthesiologists (ASA) grade, and comorbidities including hypertension, diabetes, cardiac disease (previous myocardial infarction, congestive heart failure, angina pectoris, arrhythmia, and cardiac valvular disease), cerebrovascular event, thyroid, kidney, liver disease, and pulmonary disease (asthma and chronic obstructive pulmonary disease). The factors inducing revision TKAs were categorized except for septic cause: aseptic loosening, component malposition, stiffness, and instability. Polyethylene wear was included in aseptic loosening [11]. Prosthesis type, the use of stem or augments, the cementing technique used for femoral and tibial component, and range of motion (ROM) were recorded. The prosthesis type was classified into a posterior stabilized (PS) or varus-valgus constrained (VVC) system. The use of stem or augment was evaluated on femoral and tibial sides. Postoperative radiographic variables evaluated include alignment, the level of joint line, posterior condylar offset (PCO), and Insall-Salvati ratio for patella height. The joint line was measured from the distal part of the lateral femoral condyle to the top of the fibular head apex as described by Figgie et al. [12]. Patella height was assessed according to the Insall-Salvati ratio, the ratio between patellar tendon length and patella length. Patella baja was considered when an Insall-Salvati ratio was < 0.80 while patella alta was considered when the ratio was greater than 1.20 [13]. PCO was measured as the thickness of posterior condyle projecting posteriorly to the tangent of the posterior cortex of the femoral shaft as described by Bellemans et al. [14]. The thigh and shin were assessed for the end-of-stem tip pain [8]. The location of the pain was confirmed by the patient marking the legs directly [8].

PROMs were evaluated by an independent investigator based on the Western Ontario and McMaster Universities Osteoarthritis Index (WOMAC) score [15] and patient satisfaction. Patients’ satisfaction was evaluated using patient satisfaction items of New Knee Society Score [16], a newly developed and validated self-reported inventory for evaluating patient satisfaction. It consisted of a 5-item (sitting, lying, getting out of bed, light household duty, and leisure recreation) questionnaire. Each item was graded on a 5-point Likert scale ranging from 0 to 8 points (8 = very satisfied, 6 = satisfied, 4 = neutral, 2 = dissatisfied, and 0 = very dissatisfied). The satisfaction score of the new KSS ranged from 0 to 40, with 0 representing the worst score and 40 the best score. A score of 20 of 40 points was considered neutral. Therefore, patients were categorized into satisfied and dissatisfied groups based on 20 points of satisfaction score using the New Knee Society Score [16].

### Statistical methods

Data were compared between patient groups exposed to the hybrid and the modified hybrid cementing techniques. Linear variables were analyzed using Kolmogorov-Wilcoxon test for nonparametric date while dichotomous variables were analyzed using chi-square test or Fisher’s exact test where appropriate for two independent samples. Descriptive analyses were based on frequencies and percentages for dichotomous variables, and the mean and standard deviation for linear variables. Multivariable logistic regression analysis was used to assess the independent effects of predictors by adding backward substitution factors identified as significant in univariate analysis (*p* value < 0.1). Odds ratios were reported for significant variables. All statistical analyses were performed using SPSS ver. 21.0 program (SPSS Inc., Chicago, IL, USA). A *p* value < 0.05 was considered statistically significant.

## Results

No significant difference was detected in patient or surgical factors between the two groups (Table 1). Preoperatively, there was no significant difference in WOMAC subscores between the two groups (Table 2).

**Table 1:** Patient demographics involving the hybrid and modified hybrid cementing techniques

	Hybrid (***n*** = 33)	Modified hybrid (***n*** = 29)	***P value***
**Age (years)**	67.9 (10.4)	69.9 (8.7)	0.429
**Gender (female, %)**	30 (90.9%)	28 (96.6%)	0.616
**BMI (kg/m**^**2**^**)**	25.4 (3.6)	26.6 (3.7)	0.193
**ASA grade**			
1	1 (3.0%)	1 (3.4%)	0.153
2	28 (84.8%)	28 (96.6%)	
3	4 (12.1%)	0 (0%)	
**Specific comorbidities**			
Hypertension	23 (69.7%)	16 (57.1%)	0.423
Diabetes	5 (15.2%)	6 (20.7%)	0.741
Cardiac disease	8 (24.2%)	3 (10.3%)	0.194
Cerebrovascular event	2 (6.1%)	0 (0%)	0.494
Thyroid disease	4 (12.1%)	0 (0%)	0.116
Kidney disease	2 (6.1%)	0 (0%)	0.494
Pulmonary disease	3 (9.1%)	6 (20.7%)	0.283
Liver disease	1 (3.0%)	1 (3.4%)	0.926
**Tourniquete time (min)**	96.3 (14.6)	91.0 (18.9)	0.219
**Joint line**	17.0 (6.8)	19.1 (8.0)	0.265
**Posterior condylar offset**	27.7 (4.8)	28.3 (3.9)	0.646
**Insall-Salvati ratio**	1.0 (0.3)	1.0 (0.3)	0.862
**Preop HKA angle**	Varus 8.1 (9.3)	Varus 8.2 (8.2)	0.945
**Postop HKA angle**	Varus 3.6 (4.2)	Varus 3.9 (3.1)	0.779
**Preop flexion contracture (FCf)**	2.4 (8.0)	7.3 (12.7)	0.078
**Preop further flexion (FF)**	113.1 (23.6)	103.2 (28.3)	0.150
**Postop flexion contracture (FC)**	0.8 (2.3)	0.9 (3.1)	0.921
**Postop further flexion (FF)**	112.8 (23.8)	108.1 (22.2)	0.445
**Reason of revision**			0.287
Aseptic cause			
Loosening	27 (81.8%)	28 (96.6%)	
Malposition	3 (9.1%)	1 (3.4%)	
Stiffness	1 (3.0%)	0 (0%)	
Instability	2 (6.1%)	0 (0%)	
**Prosthesis type**			0.609
PS	13 (39.4%)	14 (48.3%)	
VVC	20 (60.6%)	15 (51.7%)	
**Femoral augment (yes, %)**	16 (48.5%)	18 (62.1%)	0.317
**Tibial augment (yes, %)**	20 (60.6%)	20 (69.0%)	0.379

*BMI* body mass index, *ASA* American Society of Anesthesiologists, *HKA* hip-knee-ankle angle, *PS* posterior-stablized, *VVC* valgus-varus-constrained

The values are presented as the mean and standard deviation

**Table 2:** Preoperative and postoperative clinical scores

	Preoperative			Postoperative (2 years)		
	Hybrid (***n*** = 33)	Modified hybrid (***n*** = 29)	***P*** value	Hybrid (***n*** = 33)	Modified hybrid (***n*** = 29)	***P*** value
**Total WOMAC†**	64.6 (16.4)	64.7 (13.7)	0.985	21.0 (11.0)	12.4 (6.7)	0.007
**Pain**	12.3 (3.0)	13.3 (3.0)	0.244	3.9 (3.1)	1.9 (1.8)	0.027
**Stiffness**	4.3 (2.5)	3.7 (2.2)	0.349	1.3 (1.3)	1.1 (0.8)	0.571
**Function**	47.9 (12.5)	47.6 (11.1)	0.933	16.4 (8.4)	9.4 (5.1)	0.005

The values are presented as the mean and standard deviation

^†^Western Ontario and McMaster Universities (WOMAC) score

Postoperatively, at 2 years, the modified hybrid cement group included a lower percentage of patients with shin pain (3.4% vs. 24.2%, *p* = 0.029) than the group of patients exposed to hybrid cementing technique. The modified hybrid group also carried a lower proportion of patients with thigh pain without any statistical significance (3.4% vs. 15.2%, *p* = 0.201). The modified hybrid group showed significantly better subscores for pain and function and total WOMAC scores at 2 years postoperatively compared with the group treated with the hybrid cementing technique (all *p* < 0.05) (Table 2).

Overall satisfaction showed that 75.8% (*n* = 47) of patients were satisfied with revision TKAs while 24.2% (*n* = 15) claimed that they were dissatisfied. No significant association was found between femur and tibia augmentation based on augments, and the thigh or shin pain (all *p* > 0.05). Patients treated with modified hybrid cementing showed higher rates of satisfaction compared with patients treated with hybrid cementing strategies (93.1% vs. 60.6%, *p* = 0.003). The modified hybrid approach resulted in significantly superior scores of total satisfaction (30.7 vs. 23.3, *p* = 0.002) (Table 3).

**Table 3:** Comparisons of the end-of-stem tip pain and satisfaction scores based on the cementing technique

	Hybrid (***n*** = 33)	Modified Hybrid (***n*** = 29)	***P value***
**Satisfaction (yes, %)**	20 (60.6%)	27 (93.1%)	0.003
**Satisfaction score**			
**Sitting**	4.8 (2.3)	6.3 (1.8)	0.006
**Lying**	5.2 (1.8)	6.3 (1.8)	0.016
**Getting out of bed**	4.5 (2.4)	6.4 (1.7)	0.001
**Light house hold duty**	4.4 (2.3)	6.0 (1.4)	0.002
**Leisure recreation**	4.4 (2.1)	5.6 (1.5)	0.012
**Total**	23.3 (10.0)	30.7 (7.5)	0.002

The values are presented as the mean and standard deviation

Multivariate logistic regression analysis showed that the odds of satisfaction after revision TKAs increased 32.686-fold (*p* = 0.004) in patients without end-of-stem tip pain in the shin, and 9.261-fold (*p* = 0.027) in patients treated with the modified hybrid cementing technique (Table 4).

**Table 4:** Results of multivariate analysis of risk factors predicting satisfaction after revision total knee arthroplasty

	Odds ratio	95% CI	***P value***	Adjust odds ratio^**†**^	95% CI	***P value***
**End-of-tip pain (Shin)**			< 0.001			0.004
**No**	Reference			Reference		
**Yes**	52.571	5.677–486.830		32.686	2.969–359.874	
**Cementing**			0.008			0.027
**Modified hybrid**	Reference			Reference		
**Hybrid**	8.775	1.777–43.335		9.261	1.283–66.861	

^†^Adjusted for age, gender, body mass index, ASA score, and specific comorbidities (hypertension, diabetes, cardiac disease, cerebrovascular event, thyroid disease, kidney disease, lung disease, liver disease)

Three cases of wound dehiscence were observed along with one case of superficial wound infection involving the surgical site. None of the cases showed deep periprosthetic infection during postoperative follow-up.

## Discussion

The most important finding of this study was that the modified hybrid cementing technique significantly reduced the end-of-stem tip pain in the shin and enhanced patient satisfaction compared with the hybrid cementing technique.

The use of intramedullary stem for fixation stability and proper alignment in revision TKA is widely accepted among orthopedic surgeons, although the stem fixation approach is still disputed [6, 7]. Augmentation and stem extension are needed for severe bone defects. A short stem (metaphyseal fixation) is usually used with full cement. However, it is unreasonable to use a short stem in the case of severe non-contained bone defects. Thus, a long stem is needed for diaphyseal fixation [4]. The two methods for long stem fixation include cemented and cementless techniques. Cementless stem fixation is part of the hybrid cementing technique [6, 7]. Although studies do not reveal any significant differences in stability or durability between the two methods, the methods have advantages and disadvantages [7]. The full cementation technique provides excellent immediate fixation, increased flexibility in implant placement, reduced micromotion, and facilitates antibiotic delivery in cases of infection [17–19]. In addition, the cemented stem provides better load transfer to the diaphysis compared with cementless press-fit stems [20]. Finite analysis of the femoral side suggests that the cemented stem reduces load up to 58% whereas the cementless press-fit stems reduce it only to 18%. On the tibial side, the amount of load transfer distally with cemented stems are much better (24%) than in cementless press-fit (6%) [20]. Despite these advantages, the cemented stems are difficult to remove during an infection [18]. Therefore, we devised a novel cementing technique known as the modified hybrid cementing technique, with all the advantages associated with both types of cementing.

In this study, the end-of-stem tip pain in the shin was a factor significantly related to patient dissatisfaction after revision TKAs. Although the cause of end-of-stem tip pain after revision TKA is not proven, the following factors have been implicated: micromotion at the bone-prosthesis interface, excessive stress transfer to the surrounding bone because of large differences in Young’s modulus of elasticity between the stem tip and the bone at the point of contact, and stress shielding leading to bone resorption of the tibial tray resulting in movement to the stem tip [21–24]. Barrack et al. [8] have reported that patients with pain at the end of press-fit stem tip are significantly dissatisfied with surgical outcomes and pain relief. When using the hybrid cementing technique, the end-of-stem tip pain is closely related to patient dissatisfaction [8]. On the other hand, no patient was dissatisfied with the outcome after operation, although patients who underwent revision TKA with cemented stem developed end-of-stem tip pain at a similar rate [8]. The authors reported that the end-of-stem tip pain in the cemented stem may be considered as less consequential clinically. However, it is difficult to arrive at a definitive conclusion because a meaningful comparison is difficult due to the challenges associated with the relatively small number of patients with fully cemented stems and the larger population of patients with press-fit stem. In the present study, the use of the modified hybrid cementing technique decreased the patient’s pain at the end-of-stem tip and improved patients’ satisfaction, which may be explained by less micromotion, stress shielding, and low modulus elasticity in the cemented stem. The removal of the stem is also expected to be easier than that of the fully cemented stem, although it has not been actually removed [17, 19, 20].

End-of-stem pain is commonly reported with press-fit stem revision TKAs. However, its incidence rate varies among clinical studies [9, 25]. In addition, most studies failed to distinguish between knee pain and shin pain [9, 25]. The end-of-stem pain was reported in 11% femorally and 14% tibially in press-fit diaphysis engaged stem with a minimum of 2-year follow-up. In addition, 19% of patients with cemented tibial stems also reported end-of-stem pain [8]. Albino et al. [26] have also reported localized pain at the end of the stem in 9.4% femorally and 21.9% tibially with a mean follow-up of 2.6 years. Mihalko et al. [27] have reported that more than 16% (20 of 120 patients) manifest end-of-stem pain on the tibial side and 0% femorally. In the present study, such pain occurred in 9.7% (6 of 62 patients) femorally and 14.5% (9 of 62 patients) tibially in press-fit stems. Our results were comparable to those of previous studies mentioned above. On the other hand, we observed that end-of-stem tip pain was reduced following modified hybrid cementing intervention (from 24.2 to 3.4% in the shin and from 15.2 to 3.4% in the thigh). The incidence of pain in the thigh and the shin was decreased; however, the only decrease in shin pain was statistically significant.

Although the overall satisfaction rate of our revision TKA patients was 75.8%, the satisfaction rate of hybrid cementing technique group was only 60.6%. Baker et al. [11] reported the satisfaction rate of 66% in 797 aseptic revision TKAs, which was similar to that of our hybrid cementing technique group of patients. In their study, revision cases for stiffness had worst satisfaction rate of 47% [11]. Satisfaction rate after revision can be varied according to the cohort. We excluded septic revision cases to avoid confounding factors. Most of patients in this study were loosening cases who needed augmentation blocks and long stems during revision. And the end-of-stem tip pain was associated with patient dissatisfaction after revision.

Our study had several limitations. First, the demographic characteristics of the present study population such as the preponderance of female patients undergoing revision TKA should be considered when comparing with other populations comprising only Korean patients [28]. Nevertheless, the characteristics of daily activities and lifestyle in the Korean population including more frequent squatting and kneeling might shed further light for comparison of high-flexion activities [28]. Second, inherent limitations resulting from the retrospective study design based on a single institutional study include selection bias and heterogeneity in preoperative conditions between groups, which might have influenced our results. Third, two cementing techniques were performed during different time periods. Improvement in surgical skill might have influenced postoperative outcomes. However, because of the extensive experience (10 years) of the surgeon with revision TKAs, such temporal variation in surgical skills during the two cementing methods may not have had a significant influence on the postoperative outcomes. Finally, the present study might have been statistically underpowered with respect to responses received to all relevant questions. The likelihood of decreased thigh pain appeared to be sufficient. However, the decrease was not statistically significant because of the relatively small number of patients. Therefore, larger prospective studies are required in the future.

## Conclusion

Our novel technique of stem fixation in revision TKAs using a modified hybrid cementing technique reduced the end-of-stem tip pain in the shin and increased patients’ satisfaction postoperatively. Therefore, the modified hybrid cementing technique could be more effective option to reduce end-of-stem tip pain and enhance patient satisfaction following revision TKAs. Further research is needed to support our findings.

## Data Availability

The datasets used and analyzed during the current study available are from the corresponding author upon reasonable request.

## Abbreviations

TKA: Total knee arthroplasty
PROMs: Patient-reported outcome measures
BMI: Body mass index
ASA: American Society of Anesthesiologists
ROM: Range of motion
PS: Posterior stabilized
VVC: Varus-valgus constrained
PCO: Posterior condylar offset
WOMAC: Western Ontario and McMaster Universities Osteoarthritis Index
